# Copper-/Zinc-Doped TiO_2_ Nanopowders Synthesized by Microwave-Assisted Sol–Gel Method

**DOI:** 10.3390/gels9040267

**Published:** 2023-03-23

**Authors:** Luminița Predoană, Gabriela Petcu, Silviu Preda, Jeanina Pandele-Cușu, Simona Viorica Petrescu, Adriana Băran, Nicoleta G. Apostol, Ruxandra M. Costescu, Vasile-Adrian Surdu, Bogdan Ştefan Vasile, Adelina C. Ianculescu

**Affiliations:** 1 Institute of Physical Chemistry “Ilie Murgulescu” of the Romanian Academy, 202 Splaiul Independenței, 060021 Bucharest, Romania; 2 National Institute of Materials Physics, Atomiștilor 405A, 077125 Măgurele, Romania; 3 Department of Science and Engineering of Oxide Materials and Nanomaterials, Faculty of Chemical Engineering and Biotechnologies, “Politehnica” University of Bucharest, 1–7 Gh. Polizu, 011061 Bucharest, Romania

**Keywords:** copper-/zinc-doped TiO_2_ powders, microwave-assisted sol–gel method, thermal behavior, photocatalytic activity

## Abstract

Using the microwave-assisted sol–gel method, Zn- and Cu-doped TiO_2_ nanoparticles with an anatase crystalline structure were prepared. Titanium (IV) butoxide was used as a TiO_2_ precursor, with parental alcohol as a solvent and ammonia water as a catalyst. Based on the TG/DTA results, the powders were thermally treated at 500 °C. XRD and XRF revealed the presence of a single-phase anatase and dopants in the thermally treated nanoparticles. The surface of the nanoparticles and the oxidation states of the elements were studied using XPS, which confirmed the presence of Ti, O, Zn, and Cu. The photocatalytic activity of the doped TiO_2_ nanopowders was tested for the degradation of methyl-orange (MO) dye. The results indicate that Cu doping increases the photoactivity of TiO_2_ in the visible-light range by narrowing the band-gap energy.

## 1. Introduction

Environmental pollution constitutes a problem around the world, and scientists are working to achieve new or improved methods and materials to reduce such pollutants. The photocatalytic degradation of pollutants in the environment or water using solar radiation is the most used method today [1,2,3].

Nanosized TiO_2_-based materials are known as the most important photocatalyst for environmental applications. These materials are intensively studied for their advantages such as their nontoxicity, higher activity, lower price, and chemical and photoresist properties [4,5,6]. Among the three polymorphs (anatase, brookite, and rutile) of TiO_2_, anatase has the best photocatalytic activity [7,8].

A major influence on the properties of the final product is the preparation method. The current trend is to replace methods that use mechanical force (high-energy consumption and long preparation time) with gentle chemical methods (soft chemistry). By using soft chemical methods, a better control over the purity and stoichiometry of the studied metal oxides can be achieved [8,9,10].

Undoped or doped titanium-dioxide powders were obtained by different methods. Among them, the most frequently used methods in the liquid phase are: the sol–gel process [11,12,13,14,15,16], hydrothermal methods [8,17], coprecipitation [18], hydrothermal-assisted sol–gel method [19], microwave-assisted hydrothermal method [20,21], ultrasound-assisted sol–gel [18,22], and microwave-assisted sol–gel [19,23,24,25].

The sol–gel method presents advantages such as: synthesis at low temperatures and the use of simple and accessible technological equipment, the realization of materials with a clearly defined arrangement that can be controlled by the type of precursors used in the synthesis, and low cost by optimizing the energy cost and creating products with different shapes [15,18].

In recent research works, the microwave-assisted sol–gel method has received special interest for the preparation of oxide materials [26,27]. Microwave synthesis combines the advantage of speed and homogeneous heating of the precursor materials by making it possible to homogeneously heat the reaction solution by using microwave irradiation during the preparation process. The micro- and macroscopic characteristics of oxide materials, such as shape and morphology, defects of the surface area, purity, particle size, and reaction kinetics were improved by using microwave irradiation [28].

In the case of the sol–gel method, the hydrolysis and polycondensation reactions are complex and occur simultaneously. By the sol–gel method, powders or gels can be obtained, depending on the pH of the solution, which can be regulated by using catalysts. By microwave irradiation of the sol–gel solution, monodisperse nanoparticles are obtained (fast nucleation in a supersaturated solution) [29].

Up to now, numerous studies have been reported TiO_2_ materials prepared with different methods and modifications such as doping, heterojunction formation, nanocomposites, etc., for photocatalytic degradation applications [30,31,32,33]. Titanium dioxide is one of the most used semiconductors in photocatalytic applications due to its advantageous properties, such as cost effectiveness, ecofriendly nature, photostability, chemical inertness, and high stability, but it has a high band gap-energy value (around 3.2 eV) that limits its use only to the UV range [34]. Increasing TiO_2_ activity efficiency under the visible light range is one of the most desired improvements, and a facile way to accomplish that is by TiO_2_ modification with metal or nonmetal elements. Thus, intermediate energy levels are created by the dopants which reduce the band gap of TiO_2_ [35]. Furthermore, a better separation of electron–hole pairs is obtained by providing new sites that capture photogenerated electrons, leading to an increase in photocatalytic efficiency [36]. The effective mass of photogenerated electron–hole pairs, as well as the mobility and diffusibility of the photoexcited charge carriers, can be determined by the dopant and vacancy band shapes (charge-separation efficiency) [37]. Metals, such as Cu and Zn, are suitable for TiO_2_ doping in order to improve photocatalytic performance [34,38,39], presenting interest in this field due to their relative abundancy and low costs [34,40]. Although there are many reports in the literature regarding TiO_2_ doped with copper or zinc, there is still interest in optimizing the synthesis method in order to obtain materials with improved properties. Compared to the other synthesis methods (Table 1), the microwave-assisted sol–gel route has advantages over several properties of synthesized materials such as small size and homogeneity of the particle, high crystallinity, controlled morphology, and high purity [41]. All these properties of the materials obtained, controlled by choosing the optimal synthesis conditions, ultimately influenced the photocatalytic activity, as can be observed in Table 1.

The aim of this work was to obtain photocatalytic materials with improved properties that would be active under visible light for the degradation of organic pollutants from wastewater by a simple and cost-effective method. Thus, in the present paper, Cu- and Zn-doped TiO_2_ powders were prepared by the microwave-assisted sol–gel method in a basic medium. The influence of the synthesis method and dopant on the structure and the photocatalytic properties of these materials were evaluated. The TiO_2_-based nanopowders were used for the degradation of methyl-orange (MO) organic dye in order to evaluate the potential of these materials for environmental applications.

## 2. Results and Discussion

The samples were investigated following their thermal behavior, morphology, and structure, and their photocatalytic properties. In the case of the TiO_2_−Cu 2.0% MW sample, a white-green amorphous powder was obtained, while for the TiO_2_−Zn 2.0% MW sample, the obtained powder was amorphous and had a white color.

### 2.1. As-Prepared Samples

#### 2.1.1. SEM Results

**Figure 1:**
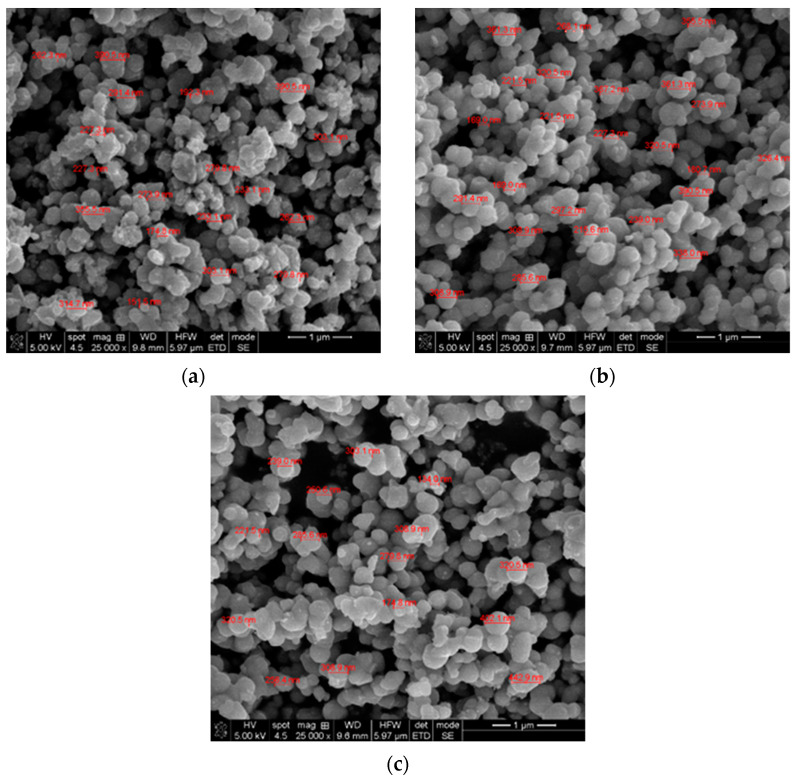
SEM micrographs of the as-prepared samples; (**a**) TiO_2_ MW, (**b**) TiO_2_−Cu 2.0% MW, (**c**) TiO_2_−Zn 2.0% MW.

SEM analysis was performed in order to investigate the morphology of the as-prepared samples, and the micrographs are shown in Figure 1.

According to the morphological observation, the size and shape of the as-prepared samples are similar, without any noticeable differences. However, in case of the TiO_2_ MW sample (Figure 1a), the nanoparticles surfaces appear to be quasi-irregular with jagged edges, while for the doped ones, the profile is well-defined.

#### 2.1.2. Thermal Behavior

**Figure 2:**
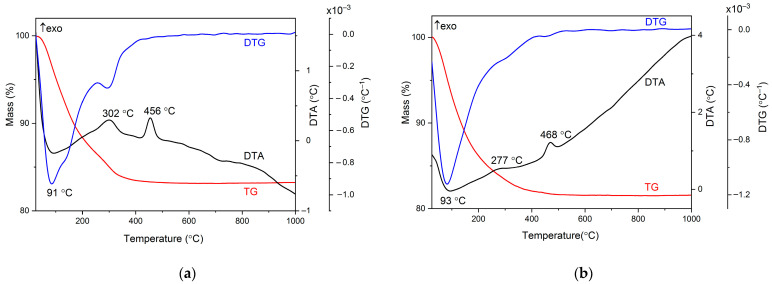
The TG (red)/DTG (blue)/DTA (black) curves of the (**a**) TiO_2_−Cu 2.0% MW sample and (**b**) TiO_2_−Zn 2.0% MW sample; heating rate was 10 °C/min, using air as carrier gas.

The thermal stability of the samples was estimated using TG/DTG/DTA analysis. The thermograms indicate the physical and/or chemical changes that occur in the samples during heat treatment. Figure 2a,b depicts the temperature-dependent mass curves of the TiO_2_−Cu 2.0% MW (Figure 2a) and TiO_2_−Zn 2.0% MW (Figure 2b) samples. During the whole measurement, the TiO_2_−Cu 2.0% MW sample exhibited a total mass loss of −16.85%, which can be further separated into three mass-loss steps of −11.62%, −4.89%, and −0.34%. The TiO_2_−Zn 2.0% MW sample showed a total mass loss of −18.58%, corresponding to three mass-loss steps of −13.91%, −4.13%, and −0.54%. The origin of the mass-loss step below 200 °C is most probably the release of physisorbed water and alcohols from the samples. Following this fact, the thermal effect on the DTA curve, corresponding to the mass loss, had the peak located at 91 °C for the TiO_2_−Cu 2.0% MW sample, and at 93 °C for the TiO_2_−Zn 2.0% MW sample, respectively.

The second mass loss, which ranged from 200 °C to 400 °C, was most probably due to the decomposition of the residual organic groups and partial dehydroxylation of Ti(OH)_4_. The thermal effect associated with the second mass loss is an exothermic effect centered at 302 °C for the TiO_2_−Cu 2.0% MW sample, and 277 °C for the TiO_2_−Zn 2.0% MW sample, respectively, as previously reported for a similar alkoxide precursor [59,60,61]. The dehydroxylation of Ti(OH)_4_ is an event that takes place over a wide temperature range and continues up to 500 °C, leading to the transformation from an amorphous phase to crystalline anatase. The second exothermic effect, located at 456 °C for the TiO_2_−Cu 2.0% MW sample, and 468 °C for the TiO_2_−Zn 2.0% MW sample, respectively, without corresponding to mass loss on the TG curve, suggests complete dehydroxylation and anatase crystallization [62,63].

#### 2.1.3. XPS on the As-Prepared Samples

To verify the oxidation state of each element, all the sample surfaces were investigated by XPS. In the following section, we will discuss the as-prepared samples. All the core-level spectra of interest (Ti 2p, O 1s, Zn 2p, Cu 2p) were analyzed using Voigt profiles, as described in ref. [64]. The integral areas obtained by the deconvolution procedure were normed to the atomic-sensitivity factors provided by ref. [65]. The binding energies were corrected such that the C value was 284.6 eV. Aside from the photoelectron emission peaks, XPS spectra can also show the presence of Auger electrons (emitted when an outer shell electron fills the photoelectron vacancy following core ionization).

**Figure 3:**
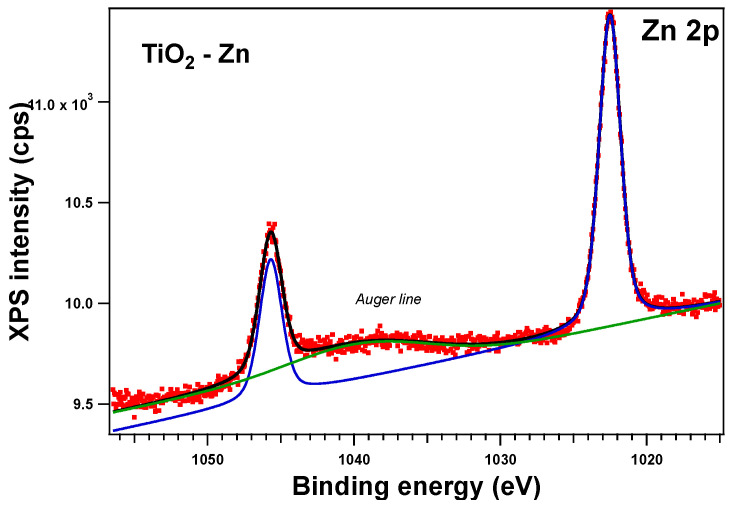
XPS spectra of the Zn 2p level of the as-prepared Zn-doped sample: red symbols for the experimental data, black line for the fit, blue line for C1, and green line for C2 (Auger line).

**Figure 4:**
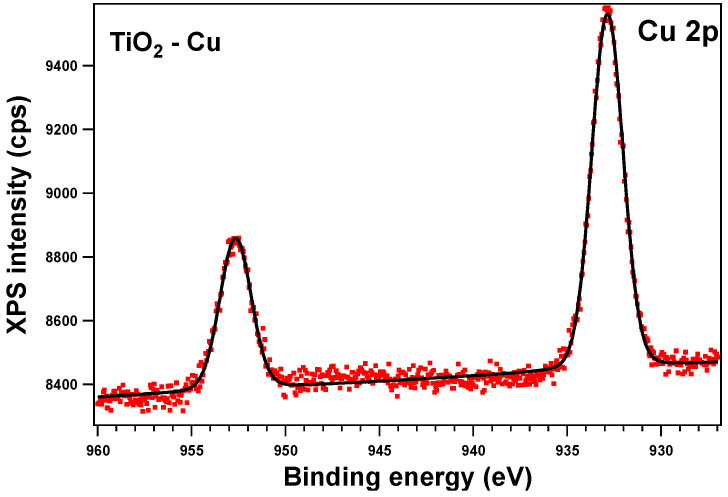
XPS spectra of the core level Cu 2p of the as-prepared Cu-doped sample: red symbols for the experimental data and black line for the one-component fit.

**Figure 5:**
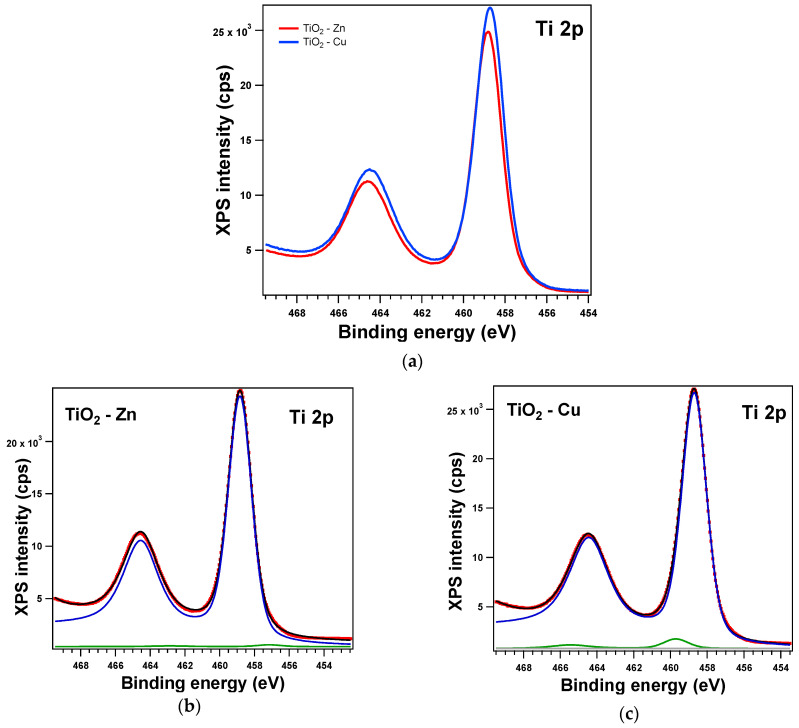
XPS spectra of the Ti 2p level: (**a**) comparison between the experimental data: red line for Zn-doped samples and blue line for the Cu-doped samples, and (**b**,**c**) the fit and deconvolutions for the Zn sample and Cu sample, respectively: red symbols for the experimental data overlayed with a black line for the fit, blue line for C1, and green line for C2.

**Figure 6:**
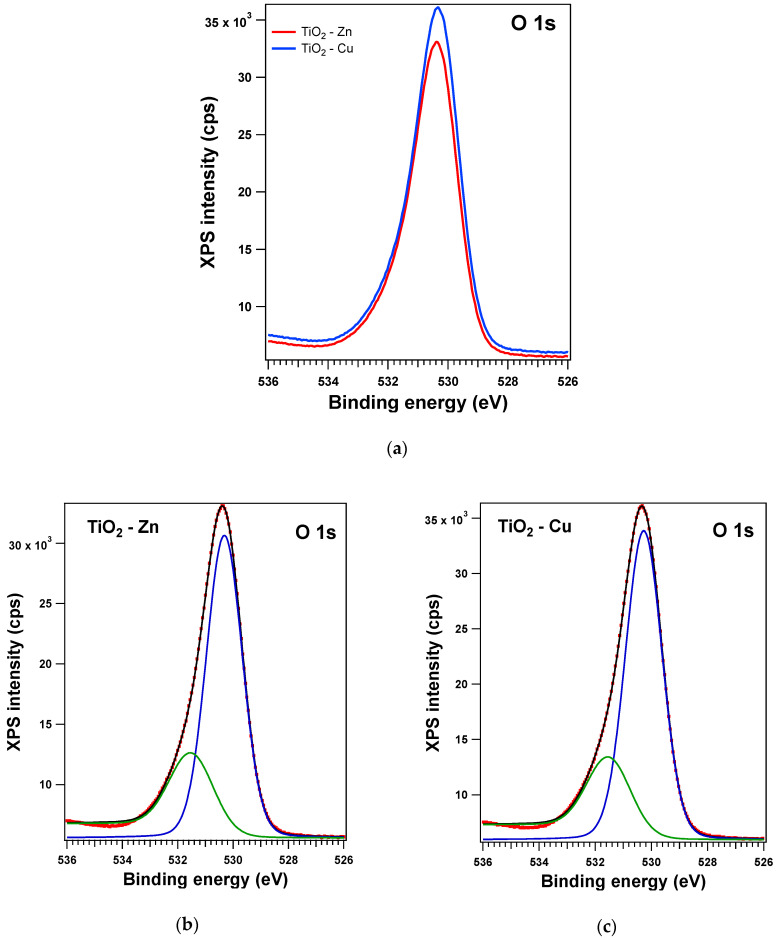
XPS spectra of the O 1s level: (**a**) experimental data for the Zn (red line)- and Cu (blue line)-doped samples and red symbols for the experimental data overlayed with a black line for the fit and deconvolutions for (**b**) the Zn sample and (**c**) the Cu sample: blue line for C1 and green line for C2.

The XPS spectra and their deconvolutions are illustrated in Figure 3, Figure 4, Figure 5 and Figure 6. The peaks for Ti 2p, O 1s, Zn 2p, and Cu 2p were attributed as described in Table 2 and revealed the presence of different species such as Ti(IV), Zn(II), and Cu(I), consistent with the database [66,67,68]. The Ti 2p doublet has a spin-orbit splitting of 5.71 eV, which supports the presence of Ti(IV). If we take into consideration the total intensity of each element, we obtain TiO_2_ with different percentages of dopants, as depicted in Table 2. 

### 2.2. Thermally Treated Samples

#### 2.2.1. SEM Results

**Figure 7:**
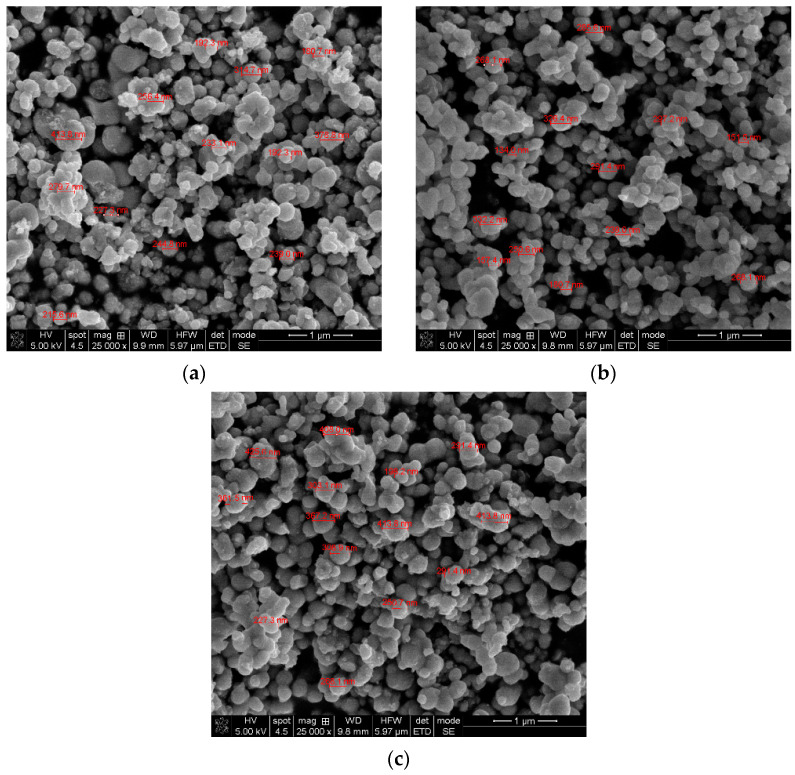
SEM micrographs of the thermally treated samples; (**a**) TiO_2_ MW, (**b**) TiO_2_−Cu 2.0% MW, (**c**) TiO_2_−Zn 2.0% MW.

The surface morphology of the thermally treated powders, obtained by SEM, are illustrated in Figure 7.

Analogous to the previously investigated untreated samples, the micrographs reveal uniformly distributed nanoparticles with the formation of aggregated clusters with similar shapes and sizes for the TiO_2_ MW sample compared to doped ones. Because of the agglomeration of the particles, no specific shapes could be determined, as previously reported [69].

#### 2.2.2. XRD Results

**Figure 8:**
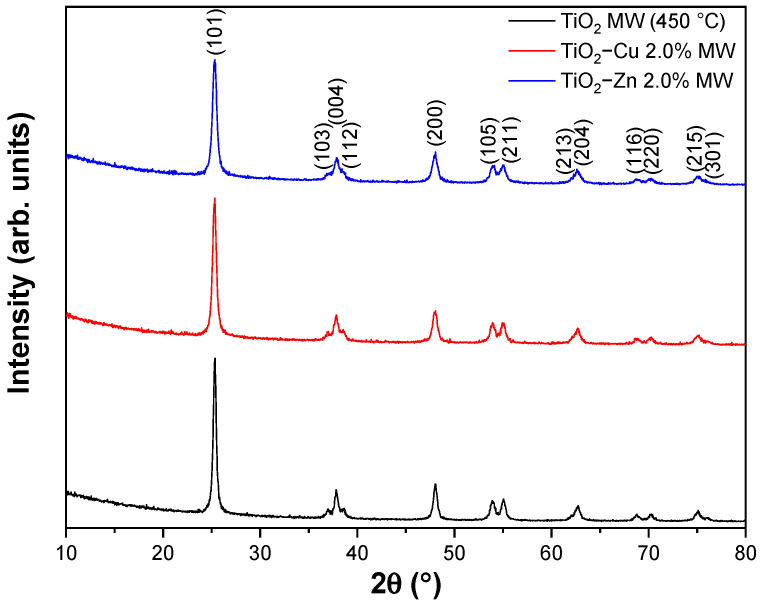
X–ray diffraction patterns of microwave-assisted sol–gel prepared samples, thermally treated (bottom—undoped TiO_2_ sample, middle—Cu-doped TiO_2_ sample, and top—Zn-doped TiO_2_ sample).

Figure 8 displays the diffractograms of the undoped TiO_2_ sample, thermally treated at 450 °C, and Zn- and Cu-doped TiO_2_ samples, thermally treated at 500 °C, respectively. All diffraction lines correspond to the TiO_2_ anatase phase, according to ICDD file no. 21−1272. No polymorphs of titanium oxide (rutile or brookite) or Cu- and Zn-based compounds were detected within the limit of the instrument. This observation suggests that the dopants enter the anatase structure or are distributed on the anatase particle surface in the form of tiny clusters [70]. To determine the impact of Cu and Zn dopants (2% molar) on the anatase structure, the lattice parameters, crystallite size, and microstrain were calculated, and the results are listed in Table 3.

According to the calculated values for the lattice parameters, no differences were noticed compared to the standard reference file (ICDD 21-1272), suggesting that Cu and Zn dopants probably substitute for Ti in the TiO_2_ host lattice. The crystallite size was influenced by the doping cation; thus, the sizes are smaller by 2 nm for Cu and 4 nm for Zn. The evolution in the mean crystallite size may be correlated with the increase in the lattice strain, where lattice strain is a measure of crystal defects, where the defects are generated by the larger ionic radius of Cu^2+^ (0.73 Å) and Zn^2+^ (0.74 Å) compared to that of Ti^4+^ (0.61 Å).

#### 2.2.3. XRF Results

The presence of the dopant elements in the sample composition was investigated by X-ray fluorescence analysis. Table 4 lists the composition in terms of elements, as well as oxides. We noticed that Cu and Zn oxides were detected in amounts close to the initial calculated composition. Other elements (C, S, Si or V) were detected as traces. Small differences compared to the initial composition could be determined by the washing procedure.

#### 2.2.4. TEM/HRTEM/SAED Investigations

**Figure 9:**
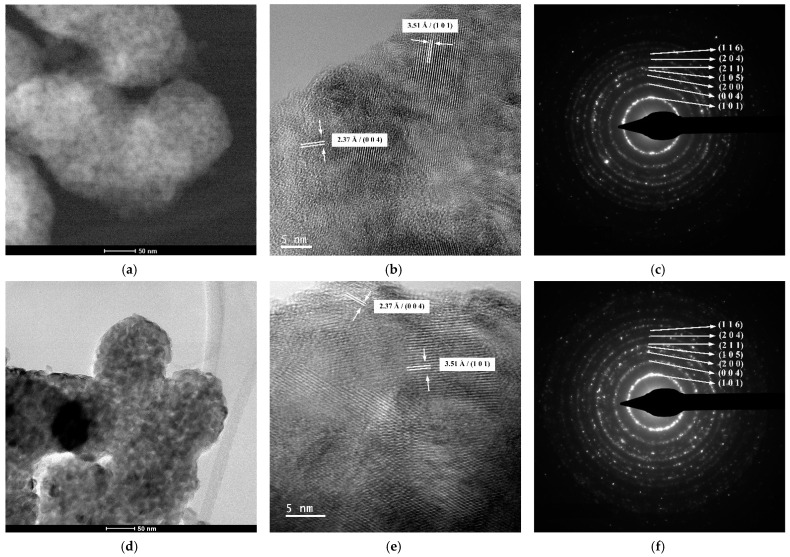
TEM images (**a**,**d**), HRTEM images (**b**,**e**), and SAED patterns (**c**,**f**) of the thermally treated samples: TiO_2_−Cu 2.0% MW (**a**–**c**) and TiO_2_−Zn 2.0% MW (**d**–**f**).

Figure 9 depicts the results of the TEM/HRTEM/SAED investigations for the thermally treated samples. Lower-magnification TEM images confirm that the quasispherical particles observed in the SEM images are aggregates of polyhedral primary nanoparticles that are nearly uniform in shape and size [70] (Figure 9a,d). Since individual nanoparticles were only spotted close to the aggregate surfaces, it is difficult to estimate the particle average size with any degree of accuracy. Nonetheless, a rough calculation shows that the values of these nanoparticles’ diameters are in the 10–20 nm range, which is in agreement with the average crystallite sizes listed in Table 3. This demonstrates that the particles in question for both analyzed powders are single-crystal. In spite of their size, the nanoparticles exhibit a high crystallinity degree, regardless of the dopant, as shown by the long-range ordered fringes inside the nanoparticles revealed by the HRTEM images in Figure 9b,e and the well-defined dashed diffraction rings made up of bright spots of the SAED patterns (Figure 9c,f).

#### 2.2.5. STEM/EDX Investigations

**Figure 10:**
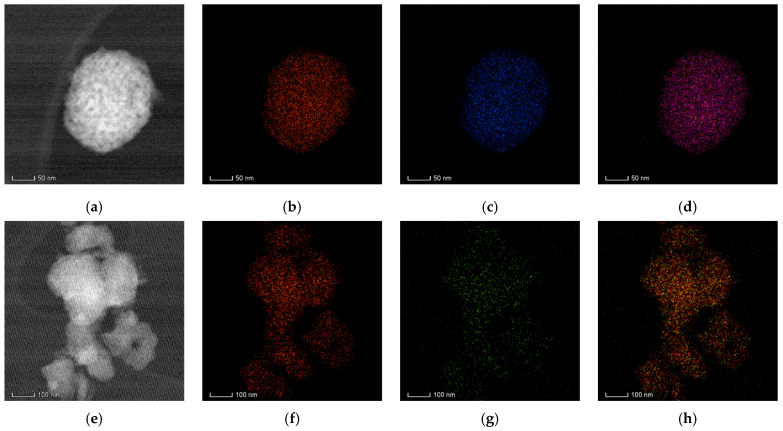
STEM image of TiO_2_−Cu 2.0% MW (**a**) and TiO_2_−Zn 2.0% MW (**e**) samples, EDX map of Ti in TiO_2_−Cu 2.0% MW (**b**) and TiO_2_−Zn 2.0% MW (**f**), Cu in TiO_2_−Cu 2.0% MW (**c**), Zn in TiO_2_−Zn 2.0% MW (**g**), Ti + Cu in TiO_2_−Cu 2.0% MW (**d**), and Ti + Zn in TiO_2_−Zn 2.0% MW (**h**).

The Ti, O, and dopant species that make up the anatase solid solutions are exclusively present, according to the EDX spectra of the Cu- and Zn-doped TiO_2_ powders, ruling out any contamination during the synthesis process (Figure 10).

Due to the consistent integration of the dopant into the host crystalline structure, the overall and elemental EDX maps (Figure 10b–d,f–h) recorded on the areas indicated by the STEM images of Figure 10a,e demonstrate a high compositional homogeneity of both powders. These results are consistent with the XRD data, revealing the presence of the single anatase phase and the absence of any segregation of any residual secondary phases.

#### 2.2.6. XPS on the Thermally Treated Samples

**Figure 11:**
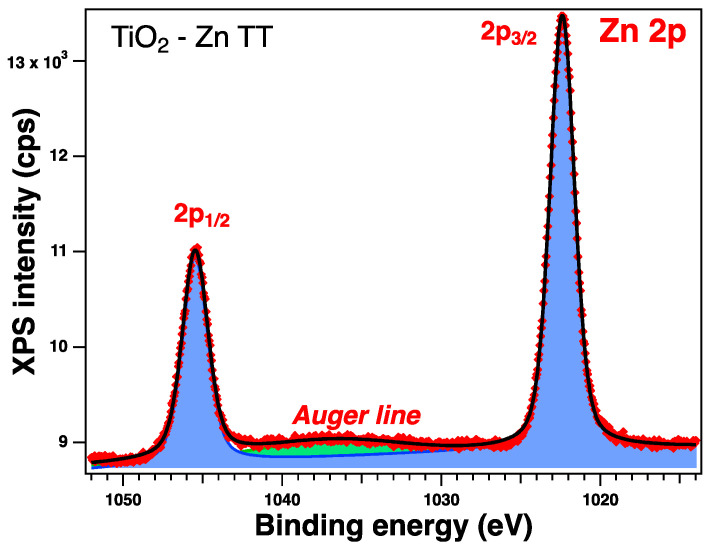
XPS spectra of the Zn 2p level of the thermally treated Zn-doped sample: red symbols for the experimental data, black line for the fit, blue line for C1, and green line for C2, which is an Auger peak.

**Figure 12:**
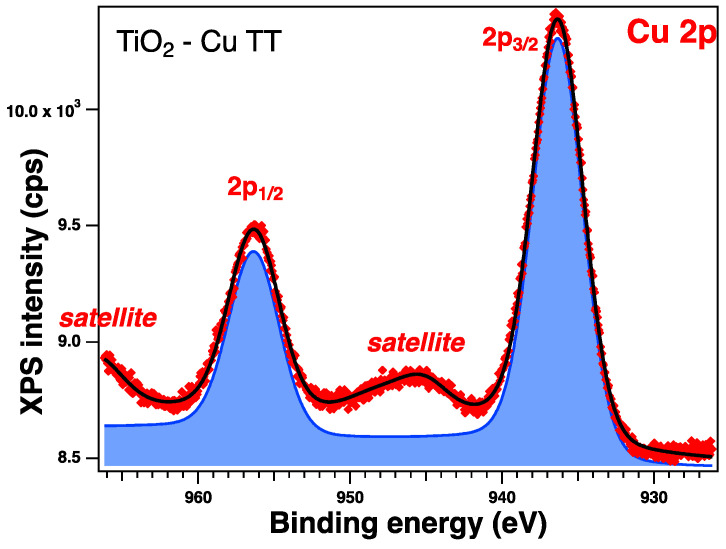
XPS spectra of Cu 2p level of the thermally treated Cu-doped sample: red symbols for the experimental data and black line for the fit with one component (blue line) and satellite peaks.

**Figure 13:**
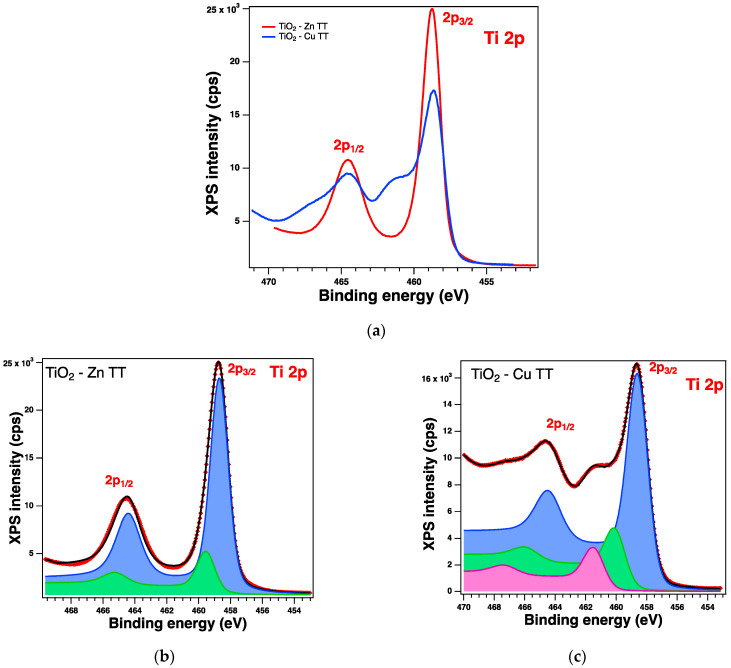
XPS spectra of the Ti 2p level for treated samples: (**a**) comparison of experimental data: red line for the Zn-doped samples and blue line for the Cu-doped samples, and (**b**,**c**) the fit and deconvolutions for the Zn and the Cu samples, respectively: red symbols for the experimental data overlayed with a black line, blue line for C1, green line for C2, and magenta line for C3 (for (**c**) only).

**Figure 14:**
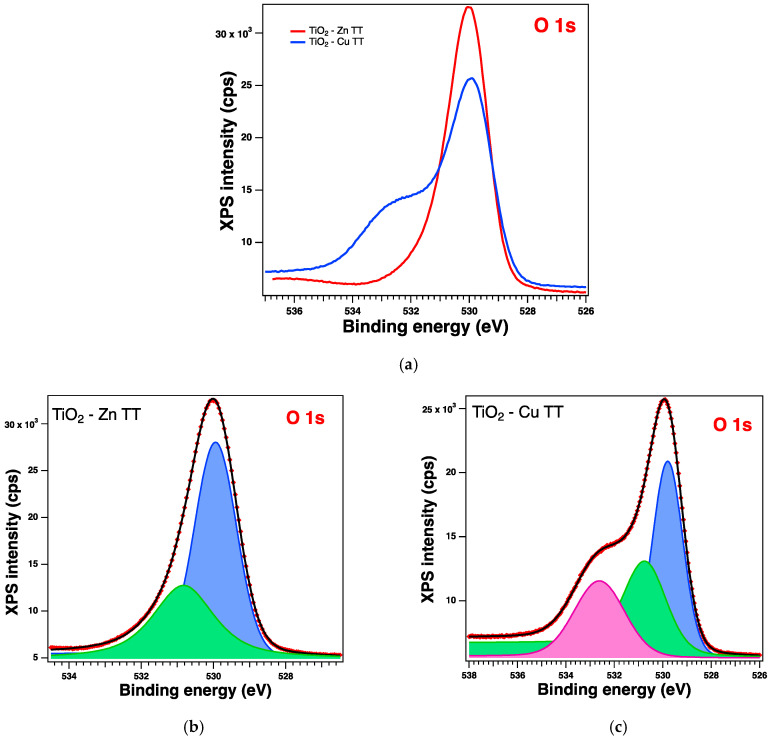
XPS spectra of the O 1s level for treated samples: (**a**) comparison of experimental data: red line for the Zn-doped samples and blue line for the Cu-doped samples, and (**b**,**c**) the fit and deconvolutions for the Zn and the Cu samples, respectively: red symbols for the experimental data overlayed with a black line, blue line for C1, green line for C2, and magenta line for C3 (for (**c**) only).

XPS measurements were also performed on the treated samples and were analyzed in the same way; see Table 5 for the relevant parameters obtained from the deconvolutions. All the core-level spectra of interest are illustrated in Figure 11, Figure 12, Figure 13 and Figure 14. The Cu 2p spectra show some additional peaks, which are satellites (peaks arising from various less-likely electron transitions). It can be observed that, in this case, there are some changes in the shape of the spectra, and the most significant one relates to the satellite peaks at ~945 eV and 967 eV, confirming the presence of the Cu(II) valence of Cu [67]; this can be assumed to result from the preparation method, as the presence of this valence was not observed when the samples were synthesized only by the sol–gel process [35].

#### 2.2.7. UV-Vis Absorption Spectra

**Figure 15:**
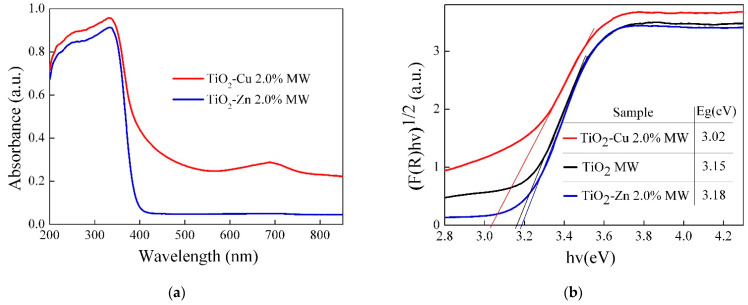
UV−Vis absorption spectra of (**a**) Cu/Zn-doped TiO_2_ MW samples and (**b**) graphic representation of the Kubelka–Munk function for indirect transitions.

The optical properties of the synthesized powders were analyzed using UV−Vis spectroscopy; the absorption spectra are shown in Figure 15a. Both doped TiO_2_ MW samples show an intense absorption band in the UV region (up to 350 nm) due to the electronic transitions O2p→Ti3d from the valence band (VB) to the conduction band (CB) of TiO_2_ [72]. A red shift of absorption band was observed for the TiO_2_−Cu 2.0% MW sample, due to the electron transition from O2p of TiO_2_ to the Cu^2+^ d-states (400–550 nm) and due to the d–d transitions of Cu^2+^ ions (600–800 nm) [73,74], indicating that doping TiO_2_ MW with copper led to an increase in visible-light-absorption capacity of the synthesized material. For the TiO_2_−Zn 2.0% MW sample, no absorption bands assigned to d–d transitions were observed, due to the complete electronic configuration of incorporated Zn^2+^ species [38].

The indirect band gap energy of the samples was estimated using the Kubelka–Munk function by plotting (F(R)*hυ)^1/2^ versus photon energy hυ (eV). As shown in Figure 15b, by doping TiO_2_ MW with copper, a narrowing of the band-gap energy was noticed, from 3.15 eV to 3.02 eV. It is probably due to the new electronic levels provided by the copper species under the conduction band of titania, available to accept the photoexcited electrons from the valence band of titania. The same behavior was not observed in the case of zinc doping, which led to a slight increase in the band-gap value from 3.15 eV to 3.18 eV. It is related to the completely filled 3d^10^ electronic configuration of Zn^2+^ species compared to Cu^2+^ with 3d^9^ configuration [38].

#### 2.2.8. Photoluminescence Analysis

**Figure 16:**
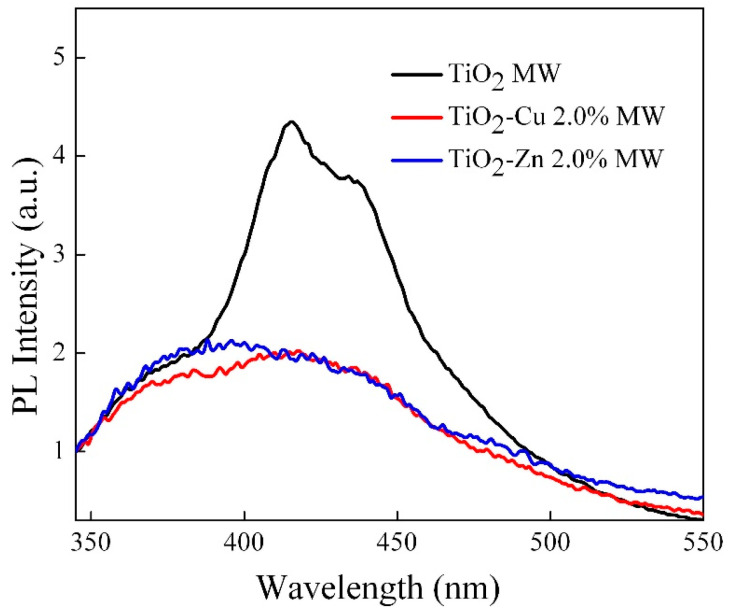
Photoluminescence spectra of doped and undoped TiO_2_ nanopowders (λ_exc_ = 320 nm).

**Figure 17:**
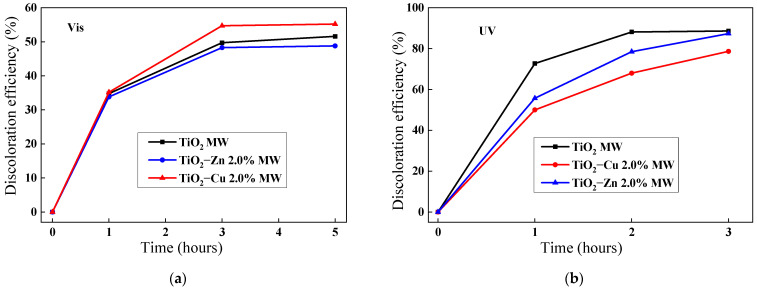
Photocatalytic activity of the samples under (**a**) visible- and (**b**) UV-light irradiation.

The photoluminescence spectra of the samples are illustrated in Figure 16. The intense signals recorded for the TiO_2_ MW sample are due to the electron–hole repairing after the return of photoexcited electrons from the conduction band to the valence band of TiO_2_. By modification with copper and zinc dopants, we noticed a quenching of the PL intensity of TiO_2_ MW, indicating the suppression of e^−^/h^+^ pair recombination by providing interband levels. This explains the better photocatalytic activity visible for the TiO_2_-Cu 2.0% MW sample with the lowest-intensity PL signal (Figure 17a).

#### 2.2.9. Photocatalysis Investigation

The photocatalytic properties of the synthesized nanopowders were evaluated in the photocatalytic degradation of methyl-orange dye under visible- and UV-light irradiation (Figure 17). The best photocatalytic activity under visible-light irradiation (Figure 17a) was observed for the copper-doped TiO_2_ sample, with a photocatalytic efficiency of 55.5% after 5 h of irradiation. This is related to the decrease in band-gap energy by doping with copper, which represents the minimum energy required for the electrons’ excitation. The presence of features attributed to Cu^2+^ and surface hydroxyl groups in the XPS spectra (Figure 14) can contribute to this photocatalytic activity. Furthermore, the presence of surface hydroxyl groups suggested by the XPS spectra (Figure 14) could improve the photocatalytic activity [75,76]. Under UV-light irradiation (Figure 17b), the synthesized materials showed high photocatalytic activity, reaching almost 90% discoloration efficiency after 3 h of irradiation.

#### 2.2.10. Identification of Reactive Species

**Figure 18:**
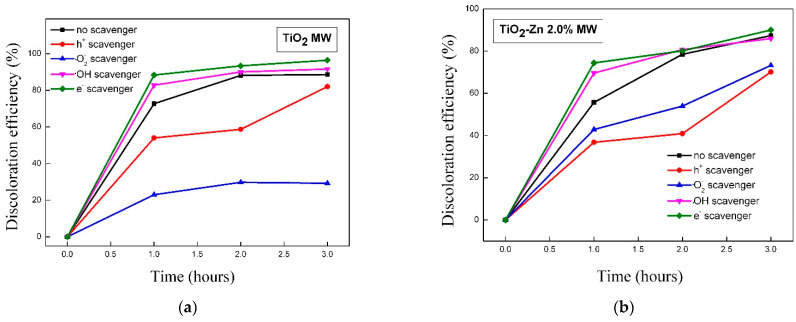

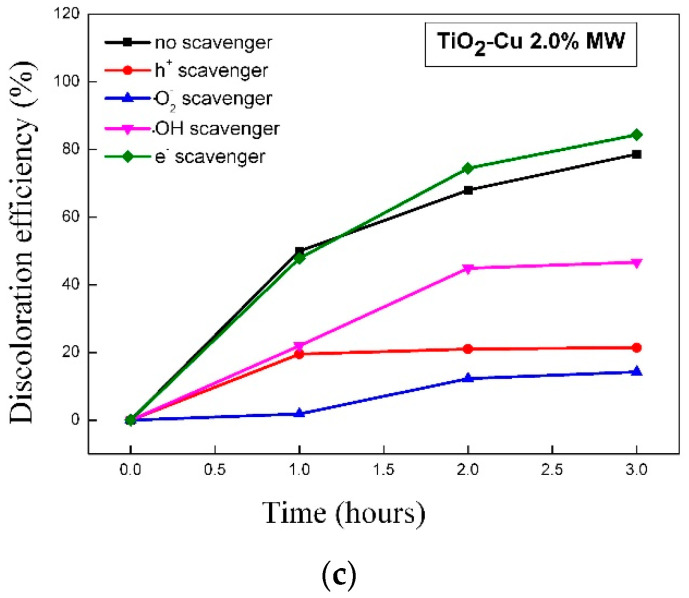
The effect of scavengers on the photocatalytic degradation of methyl orange under UV-light irradiation for (**a**) TiO_2_ MW, (**b**) TiO_2_-Zn MW, and (**c**) TiO_2_ −Cu MW samples.

In order to investigate the contribution of the main reactive oxygen species (ROS) to the MO degradation, the photocatalytic experiments were conducted in the presence of •OH, •O_2_^−^, e^−^, and h^+^ scavengers. The results are illustrated in Figure 18a–c.

The order of reactive oxygen species contribution to MO degradation on TiO_2_ MW and TiO_2_-Cu 2.0% MW photocatalysts was as follows: •O_2_^−^ > h^+^ > •OH > e^−^, indicating that the photocatalytic degradation of MO dye mainly proceeded by the attack of superoxide radicals. The significant decrease in the photocatalytic performances noticed after p-benzoquinone addition (used as •O_2_^−^ scavenger) suggests that •O_2_^−^ species have a crucial role in the methyl-orange-degradation process by the two photocatalytic materials (Figure 18a,c).

In the case of the TiO_2_-Zn 2.0% MW sample, the obtained results (Figure 18b) suggested that the photocatalytic degradation of MO was attributed especially to the holes, the order of the reactive species being the following: h^+^ > •O_2_^−^ > •OH > e^−^.

An increase in the photocatalytic activity was observed when AgNO_3_ was added into the reaction system as electron scavenger for all the samples, as a result of preventing e^−^/h^+^ recombination. In this way, there is a greater number of holes and electrons in the system, available to give rise to hydroxyl and superoxide radicals, respectively.

## 3. Conclusions

The different electron configurations of the Zn^2+^ and Cu^2+^ cations influenced the optical properties of the doped materials. By modifying TiO_2_ MW with copper, it was possible to lower the band-gap energy to 3.02 eV, which led to an increase in the photocatalytic performance in the visible range. The experimental results showed that, under visible-light irradiation, the TiO_2_−Cu 2.0% MW sample had a discoloration efficiency of 55% for MO dye after 5 h.

## 4. Materials and Methods

### 4.1. Materials

Cu- and Zn-doped TiO_2_ nanopowders were obtained using the microwave-assisted sol–gel technique. Compositions with a TiO_2_:CuO or TiO_2_:ZnO molar percentage of 98:2 were selected. Except for the microwave irradiation, the preparation method and the reagents were previously described in Ref. [35]. The solution was exposed to microwave irradiation for 10 min at 200 W in an oven operating at a frequency of 2.45 GHz with a maximum power of 2000 W. To remove the water and organic residues and obtain crystallized nanometer-sized powders, the resulting oxide powder was filtered out of the solution, washed with distilled water to remove adsorbed compounds, dried, and then thermally treated at 500 °C in the air with a plateau of 1 h and a heating rate of 1 °C/min. The composition of the solutions and the experimental conditions used are shown in Table 6.

The samples were denoted (TiO_2_−Cu 2.0% MW) and (TiO_2_−Zn 2.0%MW), and the thermally treated samples (TiO_2_−Cu 2.0% MW-TT) and (TiO_2_−Zn 2.0% MW-TT).

Our previous work [8] described the synthesis procedure for the TiO_2_ MW sample (450 °C) (noted Ti-Bu-MW).

**Figure 19:**
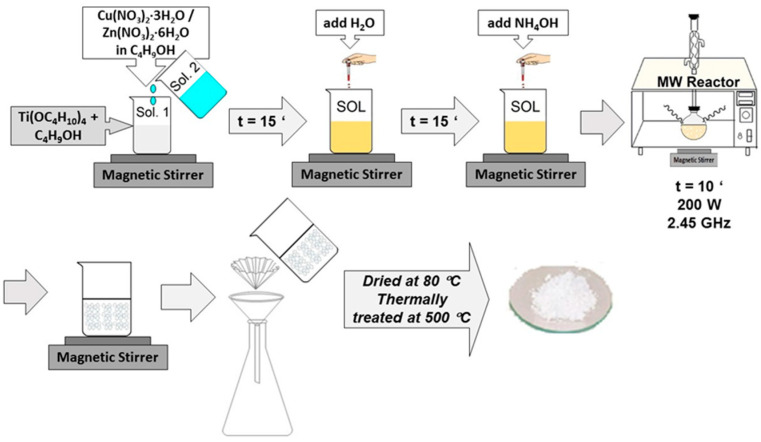
Flowchart of the methodology used for the sample preparation.

Figure 19 depicts a flowchart of the methodology used for sample preparation. Based on the TG/DTG/DTA results, the thermal treatment was determined.

In order to investigate the contribution of the main important reactive oxygen species (ROS) to the photocatalytic degradation of MO, scavenger studies were conducted. For these experiments, commonly applied quencher molecules (0.1 mmol) were used for holes (potassium iodide, KI, Merck), electrons (silver nitrate, AgNO_3_, Merck), hydroxyl (ethanol, C_2_H_5_OH, Merck), and superoxide radicals (p-benzoquinone, C_6_H_4_O_2_, Merck).

### 4.2. Methods

Thermogravimetric and differential thermal analysis (TG/DTA), using Mettler Toledo TGA/SDTA 851^e^ (Greifensee, Switzerland) equipment, were used to assess the thermal behavior of the as-prepared samples in open Al_2_O_3_ crucibles and in flowing-air environments. The heating rate was 10 °C/min, and the maximum temperature was set to 1000 °C.

An FEI Quanta 3D FEG microscope (FEI, Brno, Czech Republic) operated at a 10 kV accelerating voltage was used to capture SEM micrographs. The uncoated specimens were placed on conductive carbon tape and scanned in high-vacuum mode.

The surface of the samples was investigated by the X-ray Photoelectron Spectroscopy (XPS) measurements performed in a SPECS Multimethod Surface Analysis System, with a PHOIBOS 150 hemispherical analyzer, using Al Kα (1486.74 eV) radiation produced by a monochromatic X-ray source XR50M at operating power of 250 W (12.5 kV × 20 mA). The base pressure in the analysis chamber was at least 1.1 × 10^−8^ mbar. For charge compensation, we used a SPECS FG−40 flood-gun device, using an electron beam of 0.1 mA and 1 eV energy. High-resolution core-level spectra (Ti 2p, O 1s, Zn 2p and Cu 2p) were recorded using medium-area-lens mode and a pass energy of 30 eV.

X-ray diffraction (XRD) patterns were recorded using a PANalytical Empyrean diffractometer (Malvern Panalytical, Malvern, UK) with Ni-filtered Cu K_α_ radiation (λ = 0.15406 Å). The equipment was set on theta–theta geometry, with a 1/4° divergence slit, 1/2° antiscatter slit, and 0.02° soller slit on the incident=beam side, and a 1/2° antiscatter slit mounted on PIXCel3D detector operating in 1D on the diffracted-beam side. The scan parameters were: range 10.0000–80.0107°, step size 0.0263°, and counting time per step 255 s. Phase analysis was performed using HighScorePlus 3.0.e software coupled with ICDD PDF4+ 2022 database. Determination of unit-cell parameters, average crystallite size, and microstrains was performed by Rietveld formalism, using a polynomial background with 4 parameters and a pseudo-Voigt function for line profiles.

Elements were analyzed using X-ray fluorescence (XRF). A Rigaku ZSX Primus II spectrometer (Rigaku Corp., Tokyo, Japan) with a 4.0 kW Xray Rh tube was used for the measurements. For data analysis, EZscan was combined with Rigaku SQX fundamental parameters software (standard less).

TEM/HRTEM/SAED investigations were carried out on the powders’ morphology and crystallinity using a Thermo Fisher Scientific TITAN THEMIS Ultra High-Resolution Electron Microscope (Hillsboro, OR, USA). The transmission-electron microscope was used in STEM (scanning transmission-electron microscopy) mode at 300 kV to acquire the EDX spectra and elemental maps, with a HAADF (high-angle annular dark-field) detector for imaging and a column windowless 4 Super EDX detector for elemental analysis.

An FLSP 920 spectrofluorometer was employed to record the photoluminescence spectra (PL) of the powders (Edinburgh Instruments, Livingston, UK). An Xe lamp was used as the excitation source, the excitation wavelength was 350 nm, and the spectra were recorded between 350 and 600 nm. Using a spectrofluorometer FluoroMax 4P (Horiba Jobin Yvon, Northampton, UK), the ability of the material to produce hydroxyl radicals in solution when exposed to light was assessed. This method employs terephthalic acid (TA) (5 × 10^−4^ M TA solution, prepared in aqueous NaOH solution with a concentration of 2 × 10^−3^ M), which interacts with the hydroxyl radicals generated by the photocatalytic materials during irradiation (λ_exc_ = 312 nm), yielding a highly fluorescent compound (2-hydroxyterephthalic acid).

The optical absorption spectra of powders were recorded using a JASCO V570 spectrophotometer (Tokyo, Japan). The photocatalytic activity of doped and undoped TiO_2_ was measured in terms of the discoloration of methyl orange (MO) dye. Thus, 5 mg of photocatalyst was dispersed in 10 mL of MO aqueous solution (1 × 10^−5^ M), and further, the reaction mixture was stirred in the dark for 30 min in order to allow the adsorption of MO dye molecules on the photocatalyst surface. Then, the suspension stirred at the same constant speed was irradiated for a certain period of time (300 min in the case of UV irradiation and 180 min under visible-light irradiation) in a closed box with a UV−Vis lamp at certain specific wavelengths. At regular intervals of time, we took the same aliquots of MO solution and filtered them using syringe filters with a 0.45 μm pore size, and spectrophotometrically analyzed them in order to evaluate the progress of the photocatalytic reaction. The discoloration efficiency of the samples was evaluated using the absorbance value of the maximum peak (464 nm) that corresponds to the azo bond of MO dye recorded at the beginning of the reaction and after each time interval. In the case of ROS-scavenging experiments, the procedure was the same as in a photocatalytic test, except for the addition of scavengers (0.1 mmol) to the reaction mixture.

## Figures and Tables

**Table 1:** Comparative results reported in the literature on methyl-orange photocatalytic degradation in the presence of Cu- or Zn-doped TiO_2_ obtained by different methods.

Preparation Method	Dopant Content	Irradiation-Light Type	Methyl-Orange Concentration	Degradation Efficiency, [%]	Ref.
Cu-doped TiO_2_					
sol–gel method	TiO_2_−Cu 2.0%	UV and visible light	1 × 10^−5^ M	97% UV 16% Vis	[35]
hydrothermal process (180 °C for 8 h)	0, 2, 4, 6, 8, and 10% Cu-TiO_2_	simulated sunlight irradiation (300 W Xe lamp)	20 mg/L	87.7% of MO (3 cycles, 6% Cu-TiO_2_) Photocatalytic degradation efficiencies of MO: 6% Cu-TiO_2_ > 4% Cu-TiO_2_ > 8% Cu-TiO_2_ > 2% Cu-TiO_2_ > 10% Cu-TiO_2_ > TiO_2_	[42]
TiO_2_ by sol–gel method; [Cu(OHCor)]/TiO_2_ composite by reflux	[Cu(OHCor)]/TiO_2_	visible white LED lamp of 30 W	10 ppm	31.0%@2.5 h for TiO_2_ 79.5% for [Cu(OH-Cor)]/TiO_2_ Decrease by 5.7% ([Cu(OH-Cor)]/TiO_2_ after 3 cycles	[43]
hydrothermal method (220 °C for 24 h)	Fe_3_O_4_@TiO_2_ 1 wt%, 2 wt% and 3 wt% Cu-Fe_3_O_4_@TiO_2_	sunlight illumination	20 mg/L	85%@2.5 h for MO for Fe_3_O_4_@TiO_2_ 85%@2.5 h for MO for 3 wt% Cu-Fe_3_O_4_@TiO_2_	[44]
sol–gel method	TiO_2_ Cu/TiO_2_ rFA/Cu/TiO_2_ oxide Acid- FA/Cu/TiO_2_ Base- FA/Cu/TiO_2_	UVA (λ = 365 nm); visible light (Opple, 4.5 W)	10 ppm	81.8% UV, 6.7% Vis for TiO_2_ 37.4% UV, 15.3% Vis for Cu/TiO_2_ 79% UV 58.8% Vis for rFA/Cu/TiO_2_ 100% UV and 99.1%% Vis for Base-FA/Cu/TiO_2_ 96.9% UV for Acid-FA/Cu/TiO_2_	[45]
solvothermal method	TiO_2_ 0.1 mol%Cu-TiO_2_ 0.3 mol%Cu-TiO_2_ Cu/N-TiO_2_ 0.5 mol%Cu-TiO_2_ 1 mol%Cu-TiO_2_	simulated visible light (250 W hydrogen lamp, 464 nm)	20 mg/L	94.3% after 8 cycles for Cu/N-TiO_2_ Cu/N-TiO_2_ is four times better than TiO_2_ (reaction rate constant 0.695 h^−1^)	[46]
one-step solvothermal synthesis method	TiO_2_-RGO TiO_2_-RGO-xCuO (x = 0.05, 0.075, 0.1, 0.3, 0.5%)	simulated visible light (250 W neon lamp, 464 nm)	20 ppm	94.8% after 8 cycles for TiO_2_-RGO-0.075%CuO	[47]
sol–gel method	TiO_2_ Cu/TiO_2_ (1:1 wt%) Cu/TiO_2_/FA	UVA (λ = 365 nm); visible light (Opple, 4.5 W)	5, 15, 25, or 100 ppm	54.32% for Cu/TiO_2_ (visible) 11.59% for TiO_2_ (visible) 89.53% for TiO_2_ (UV) 70.27% for Cu/TiO_2_ (UV) 96.78% for Cu/TiO_2_/FA (UV) 89.54% for Cu/TiO_2_/FA (Visible)	[48]
hydrothermal synthesis method (24 h at 200 °C)	TiO_2_ TiO_2_/Cu OPMTC (Organic porous materials-TiO_2_/Cu composite)	simulated sunlight (a); nature sunlight (b)	100 mg/L	55.8% (a), 49.1% (b) 91.8% (a), 86.5% (b) 96.3% (a), 92.6% (b)	[49]
in situ approach	AA AA-0.5Cu AA-1Cu AA-1.5Cu AA-5Cu AA-10Cu P25 P25-0.5Cu P25-1Cu P25-1.5Cu P25-5Cu P25-10Cu	UV-A (6 × 6 W fluorescence lamp, 365 nm)	1 g L^−1^	75.6% 7.1% 6.6% 14.5% 17.4% 23.1% 82.8% 37.5% 38.1% 30.5% 24.9% 39.1%	[50]
hydrothermal	Cu	UV	10 ppm	90% in 150 min	[51]
**Zn-doped TiO_2_**					
sol–gel method	TiO_2_−Zn 2.0%	UV and visible light	1 × 10^−5^ M	90% UV 30% Vis	[35]
Micro-arc oxidation, impregnation	MAO (TiO_2_) MAOZn (Zn-TiO_2_)	UV (250 W, 365 nm)	5 mg·L^−1^, 10mg·L^−1^, 15mg·L^−1^ and 20 mg·L^−1^	94% MAOZn films 90% after 10 cycles	[52]
sol–gel reflux synthesis route	Zn (3 mol %)-TiO_2_ Zn (5 mol %)-TiO_2_	UV-A (1.29 mW cm^−2^, 466 nm)	1 mg of dye in 100 mL H_2_O	95.6% for MO 99.6% for MO	[53]
sol–gel route	Ag,Zn-TiO_2_	solar simulator (100 LCL Compact Xenon Light lamp)	4 ppm	58.5% at pH 11 84.4% at pH 2.1, 2 gL^−1^ catalyst dose 93.1% at pH 4.1, 2 gL^−1^ catalyst dose Complete mineralization at 8 gL^−1^ catalyst dose within 60 and 120 min for Ag–Zn-TiO_2_	[54]
simple coprecipitation method	TZO-4 (ZnO/TiO_2_)	UV (500 W, λ max = 365 nm)	20 mg/L	99%/90 min	[55]
stearic-acid-gel method; sol–gel method	P25 (0, 0.05, 0.1, 0.3, 0.5, 1)_ste_ Zn-TiO_2_ 0.1_sol_ Zn-TiO_2_ At 400, 450, 500 and 600 °C	mercury lamp (300 W)	20 mg/l	0.1% Zn/TiO_2 ste_—best photodegradation of the dye 0.1% Zn/TiO_2 ste_ > 0.1% Zn/TiO_2 sol_ > P25 For 0.1%Zn/TiO_2 ste_ series 450 °C > 400 °C > 500 °C > 600 °C	[56]
ligand exchange reaction and with additional thermal treatment	Pure TiO_2_ metal oxide TiO_2_(-Zn) TiO_2_(-Zn)+HCl	UV light reactor (400 W high-pressure mercury lamp)	20 mg/l	Residual MO 0.799 mg/L@1h, 0.637 mg/L@2h and 0.528 mg/L@3h for metal oxide TiO_2_(-Zn) 0.859 mg/L@1h, 0.748 mg/L@2h and 0.685 mg/L@3h for pure TiO_2_ 0.742 mg/L@1h, 0.542 mg/L@2h and 0.403 mg/L@3h for TiO_2_(-Zn)+HCl	[57]
ligand exchange reaction and with additional thermal treatment	TiO_2_ nanotubes Zn(acac)_2_ assembled TiO_2_ nanotubes	UV light reactor (400 W high-pressure mercury lamp)	20 mg/L	Residual MO in Zn(acac)_2_ assembled TiO_2_ nanotubes At 300 °C 19.72 mg/L@1h, 19.08 mg/L@2 h and 18.24 mg/L@3 h At 400 °C 13.82 mg/L@1 h, 9.44 mg/L@2h and 7.02 mg/L@3 h At 500 °C 15.32 mg/L@1 h, 12.70 mg/L@2 h and 10.82 mg/L@3 h Zn ions surface-doped TiO_2_ nanotubes > Pure TiO_2_ nanotubes > pure TiO_2_ nanoparticles	[58]

**Table 2:** Binding energies (BE), atomic %, and attributions of the deconvolutions for the core levels for the as-prepared samples.

	Element		BE (eV)	% at	Interpretation
TiO_2_-Zn 2.0% MW	Ti 2p	C1	457.15	0.25	Ti(IV) vol.
		C2	458.82	32.59	Ti(IV) surf.
				32.84	
	O1s	C1	530.32	50.71	Ti(IV)
		C2	531.49	15.9	TiO_2_/TiO_x_ + Zn(II) + cont
				66.61	
	Zn 2p_3/2_	C1	1022.52	0.55	Zn(II)
					TiO_2.03_—Zn 0.55%
TiO_2_-Cu 2.0% MW	Ti 2p	C1	458.70	32.58	Ti(IV) vol.
		C2	459.71	1.26	Ti(IV) surf.
				33.84	
	O1s	C1	530.28	51.02	Ti(IV)
		C2	531.50	14.75	TiO_2_/TiO_x_ + Zn(II) + cont
				65.77	
	Cu 2p_3/2_	C1	932.86	0.39	Cu(I)
					TiO_2_—Cu 0.39%

**Table 3:** The lattice parameters, the estimated crystallite size, and the average microstrain of the samples.

Sample	Lattice Parameters		Crystallite Size, [nm]	Microstrain, [%]
	a, [Å]	c, [Å]		
TiO_2_ MW (450 °C)	3.788359 ± 0.000278	9.508230 ± 0.000739	16	0.57 ± 0.16
TiO_2_−Cu 2.0% MW	3.788145 ± 0.000340	9.504234 ± 0.000896	14	0.65 ± 0.19
TiO_2_−Zn 2.0% MW	3.790948 ± 0.000383	9.500206 ± 0.001015	12	0.75 ± 0.22
TiO_2_, anatase (ICDD 21-1272)	3.7850	9.5140	-	-

**Table 4:** Elemental composition of the analyzed samples.

Sample	Composition	Values	U.M.	Line
TiO_2_−Zn 2.0% MW	Ti	57.9936	mass%	Ti−KA
	Zn	1.6021	mass%	Zn−KA
	O	39.0359	mass%	O−KA
	C, S, Si, V (traces)	1.6684	mass%	
	TiO_2_	93.2240	mass%	Ti−KA
	ZnO	1.8994	mass%	Zn−KA
	C, S, Si, V oxides (traces)	4.8766	mass%	
TiO_2_−Cu 2.0% MW	Ti	56.7392	mass%	Ti−KA
	Cu	1.6454	mass%	Cu−KA
	O	40.3301	mass%	O−KA
	C, Si, S (traces)	1.2	mass%	
	TiO_2_	93.3325	mass%	Ti−KA
	CuO	2.0222	mass%	Cu−KA
	C, S, Si oxides (traces)	4.6453	mass%	

**Table 5:** Binding energies (BE), atomic %, and attributions of the deconvolutions for the core levels for the treated samples.

	Element		BE (eV)	% at	Interpretation
TiO_2_-Zn 2.0% TT (500 °C)	Ti 2p_3/2_	C1	458.7	24.1	Ti(IV) vol.
		C2	459.53	4.5	Ti(IV) surf.
				28.6	
	O 1s	C1	529.94	42.5	Ti(IV)
		C2	530.83	27.1	Ti(IV)+Zn(II)+cont
				69.6	
	Zn 2p_3/2_	C1	1022.39	1.8	Zn(II)
					TiO_2,43_—Zn 1.8%
TiO_2_-Cu 2.0% TT (500 °C)	Ti 2p	C1	458.6	16.7	Ti(IV)
		C2	460	4.2	TiO_x_
		C3	461.4	3.1	Ti
				24.0	
	O 1s	C1	529.79	31.5	Ti(IV)
		C2	530.71	21.8	TiO_x_+ organics
		C3	532.61	20.8	TiO_x_/OH groups [71]
				74,1	
	Cu 2p_3/2_	C1	936.3	1,9	Cu(II)
					TiO_2,22_—Cu 1.9%

**Table 6:** The composition and the experimental conditions.

Sample	Precursors	Molar Ratio			pH Sol	Experimental Conditions	
		$\frac{ROH}{\sum precursor}$	$\frac{H_{2}O}{\sum precursor}$	$\frac{catalyst}{\sum precursor}$		T (°C)	t (min)
TiO_2_−Cu 2.0% MW	Ti(OC_4_H_10_)_4_ + Cu(NO_3_)_2_·3H_2_O	36.5	3	0.003	10	60	10
TiO_2_−Zn 2.0% MW	Ti(OC_4_H_10_)_4_ + Zn(NO_3_)_2_·6H_2_O	36.5	3	0.003	10	60	10

ROH = C_4_H_9_-OH.

## Data Availability

All data are available upon reasonable request from the authors.
